# Taking the tube to the basal pole of hair cells

**DOI:** 10.7554/eLife.107824

**Published:** 2025-07-23

**Authors:** Satish R Ghimire, Thomas M Coate

**Affiliations:** 1 Department of Biology, Georgetown University Washington, DC United States

**Keywords:** ribbons, microtubules, hair cells, sensory processing, ribbon synapses, kinesin, Zebrafish

## Abstract

A stable microtubule network and kinesin motors facilitate the formation of presynaptic ribbons in sensory hair cells in the zebrafish lateral line.

**Related research article** Hussain S, Pinter K, Uhl M, Wong HT, Kindt KS. 2025. Microtubule networks in zebrafish hair cells facilitate presynapse transport and fusion during development. *eLife* **13**:RP98119. doi: 10.7554/eLife.98119.

Our eyes and ears must transmit sensory stimuli quickly and accurately. They achieve this through specialized sensory cells, such as photoreceptors in the eye and hair cells in the inner ear. Structures within these cells, known as ribbon synapses, enable these receptors to convey to the brain the duration and intensity of a stimulus with remarkable fidelity, and for as long as the stimulus is present (Matthews and Fuchs, 2010).

A ribbon synapse features a large synaptic density, the ribbon, which is located at the base or basal pole of photoreceptors and hair cells. In this area – also known as the presynaptic active zone – a ribbon is associated with a large number of vesicles containing neurotransmitters, readily available for release upon sensory stimulation (Figure 1A and B; Lenzi and von Gersdorff, 2001). Ribbons are primarily made up of a protein called RIBEYE, which facilitates the release of neurotransmitters (Schmitz et al., 2000). Early during development, small ribbon precursors are distributed throughout sensory receptor cells. These precursors later fuse to form larger ribbons that migrate to the presynaptic active zone (Michanski et al., 2019; Wong et al., 2014). However, the mechanisms and timing of ribbon maturity, as well as their subsequent migration to their destination, have long remained a mystery.

**Figure 1.**
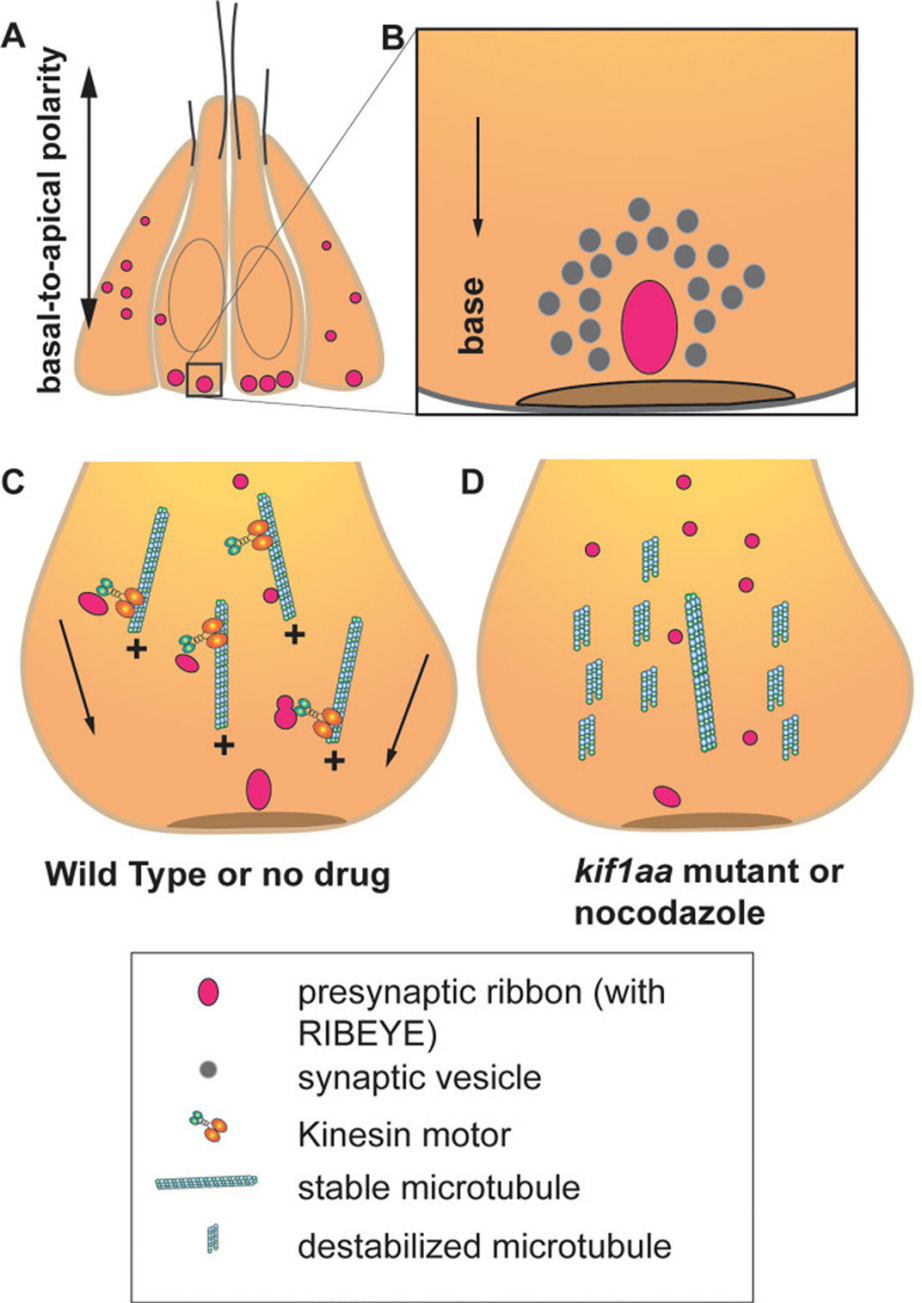
Formation of ribbon synapses in the hair cells of zebrafish. (**A**) Hair cells in zebrafish lateral-line neuromasts displaying ribbons at different stages of maturation (pink dots). At two days post-fertilization, neuromast hair cells have several small ribbons scattered throughout the cells (left and right). In mature hair cells, ribbons are bigger and confined to the base of the hair cells (center). (**B**) In mature hair cells, a ribbon is located close to the presynaptic active zone (brown disc) and is associated with a pool of synaptic vesicles containing neurotransmitter-filled vesicles (grey circles). (**C**) In hair cells, most of the microtubules are oriented with their growing plus (+) end facing the base of the hair cells. In wild-type hair cells, stabilized microtubule networks along with kinesin motor proteins (orange, turquoise structure) transport the ribbons toward the future presynaptic active zone located at the base of the hair cells (arrows). (**D**) When the microtubule network is destabilized due to nocodazole treatment or in zebrafish with the absence of the gene for the Kinesin motor Kif1aa – normal development and transport of ribbons are affected.

Now, in eLife, Saman Hussain, Katherine Pinter, Hiu-Tung Wong and Katie Kindt (all at the National Institute on Deafness and other Communication Disorders), and Mara Uhl (University Medical Center Goettingen) report new insights into early ribbon synapse formation in the hair cells of zebrafish (Hussain et al., 2025). Hussain et al. used high-resolution confocal and Airyscan imaging to study hair cells in lateral line neuromasts, sensory structures on the surface of zebrafish larvae that detect water movement. They found that small ribbon precursors use microtubules to fuse into mature ribbon synapses and to travel to their destination. These findings are not without precedent: microtubules have been previously shown to play important roles in synapse structure and function, including the transport of other factors known to be crucial at the hair cell presynaptic active zone (Parato and Bartolini, 2021).

Hussain et al. noted several key observations regarding ribbon formation and microtubule dynamics during the early stages of development. Two days after fertilization, the zebrafish larvae exhibited numerous small ribbon precursors distributed throughout immature hair cells. In contrast, more mature hair cells had fewer but larger ribbons confined to the base of the cell near the active zone. Additionally, ribbons were frequently located close to microtubules, with the growing (or plus) end of microtubules facing the base of the hair cells (Figure 1C). These observations strongly support the hypothesis that microtubules move ribbon precursors as cargo toward the base of the hair cells.

Hussain et al. also used time-lapse confocal imaging to observe the migration of a subpopulation of fluorescently tagged ribbons moving along the microtubules. When zebrafish were treated with nocodazole, a drug that destabilizes microtubules, the number of small and immature ribbons increased, resulting in fewer complete synapses (Figure 1D). This suggests that a stable microtubule network is required for the formation and movement of ribbons, as well as the formation of synapses.

Hussain et al. propose that microtubule stability over the first few hours, two days after fertilization, is crucial for ribbons to form properly. Treating zebrafish larvae with nocodazole during this narrow time window resulted in an increased number of immature ribbons. It also disrupted the directed movement and fusion of ribbon precursors, demonstrating a direct role of a stable microtubule network for ribbon synapse development.

Next, Hussain et al. addressed the hypothesis that motor proteins control the transport of ribbons toward the plus end of the microtubules in hair cells. They tested this by generating transgenic zebrafish lacking the gene for the kinesin motor protein Kif1aa, which has been linked to ribbon development in mammals (Michanski et al., 2019; Voorn et al., 2024). Indeed, these zebrafish showed increased ribbon precursors and fewer synapses (Figure 1D). However, with the help of imaging experiments in a *kif1aa* knock down model, the researchers discovered that while Kif1aa was critical for fusion events, it was dispensable for directed ribbon migration. This suggests that other kinesin proteins could be involved in directed ribbon transport.

The findings of Hussain et al. highlight an essential role of microtubules and associated motor proteins in organizing the synaptic architecture within sensory receptors across species. It remains to be seen if additional proteins are involved in ribbon transport and synapse formation, and whether they play a role in the assembly of synaptic components, such as ion channels in pre- and postsynaptic zones. Answering these questions will further our understanding of how the nervous system forms and aid drug design targeting microtubule networks for treating synapse-related disorders in visual and auditory systems.
